# A universal DNA-barcode approach to aid species identification in the family Cyperaceae

**DOI:** 10.3389/fpls.2026.1794327

**Published:** 2026-07-16

**Authors:** Prabin Bhandari, Chao Xu, Bang-Ze Li, Fu-Xing Li, Pedro Jiménez-Mejías, Shu-Ren Zhang, Shi-Liang Zhou

**Affiliations:** 1 State Key Laboratory of Plant Diversity and Specialty Crops, Institute of Botany, Chinese Academy of Sciences, Beijing, China; 2 China National Botanical Garden, Beijing, China; 3 University of Chinese Academy of Sciences, Beijing, China; 4 Department of Botany, Tribhuvan University, Kathmandu, Nepal; 5 Henan University of Science and Technology, Luoyang, China; 6 Pablo de Olavide University, Sevilla, Spain

**Keywords:** ITS, *matK*, *rbcl*, rps16, sedges, systematics, taxonomy

## Abstract

Cyperaceae, one of the ten largest plant families with a cosmopolitan distribution, presents significant taxonomic challenges due to its highly reduced and complex floral structures. These complexities make species identification through conventional morphology-based methods difficult. To address these limitations, DNA barcoding was employed for accurate species identification. This study evaluates the universality of DNA barcodes across the entire Cyperaceae family using a total of 6739 sequences. The performance of four DNA barcoding regions ITS, *matK*, *rbcL*, and *rps16*, was explored using three analytical approaches, genetic distance, sequence similarity, and phylogenetic tree methods. Among single-locus barcodes, the nuclear region internal transcribed spacer (ITS) showed higher discriminatory power than the chloroplast regions *matK*, *rbcL*, and *rps16*. Among multi-locus combinations, ITS + *matK* + *rps16* was found to be most effective. These findings provide valuable insights for improving the precise identification of Cyperaceae species. Future efforts should focus on sampling species-rich tribes such as Cariceae, Cypereae, Schoeneae, Rhynchosporeae, and Sclerieae, particularly in poorly sampled regions of Asia and Africa. Expanding these efforts is crucial for building a robust and comprehensive GenBank reference database for Cyperaceae.

## 1 Introduction

Accurate identification of species is key to the development of the catalogue of life and understanding its distribution on the planet. This task becomes particularly challenging in taxonomically complex groups (Hartvig et al., 2015). Traditional morphology-based taxonomy, while widely used and affordable, is constrained by its dependence on specialists (Yan et al., 2015a), who are becoming increasingly rare. Developing robust methodology for species identification is particularly urgent for plants, as they face growing threats from habitat loss, invasive species, and climate change. DNA barcoding has emerged as a powerful tool to address these limitations, enabling quick and reliable species-level identification across all forms of life, including animals, plants, and microorganisms (Hebert et al., 2003; Kress et al., 2005; Hubert and Hanner, 2015). This technique is especially valuable for distinguishing species that are difficult to separate based on morphological characters, offering an efficient solution for cryptic species differentiation (Mat Jaafar et al., 2012; Wang et al., 2020; Ren et al., 2024).

DNA barcodes, also known as molecular markers, are short standardized DNA regions used for species identification and phylogenetic reconstruction (Hebert et al., 2003; Kress et al., 2005; CBOL Plant Working Group et al., 2009, 2011; Hartvig et al., 2015). The selection of the marker is crucial for reliable results (Helbig et al., 2002; Hollingsworth et al., 2011; Twyford et al., 2024), as an ideal marker should be universal, reliable, phylogenetically informative, and cost effective (Kress et al., 2005; CBOL Plant Working Group et al., 2009; Xue and Li, 2011). CBOL Plant Working Group et al. (2009), on analyzing seven plastid regions (*atpF*-*atpH*, *matK*, *rbcL*, *rpoB*, *rpoC1*, *psbK*-*psbI*, and *trnH*-*psbA*) recommended the combination of *rbcL* + *matK* as the core plant barcode. Subsequently, the plastid intergenic spacer *trnH*-*psbA* and the nuclear ribosomal internal transcribed spacer (ITS) were proposed as supplementary plant barcode (CBOL Plant Working Group, 2011; Pang et al., 2012; Gere et al., 2013).

The plastid coding region *rbcL* is highly conserved and widely used in evolutionary studies due to its slow substitution rate and high amplification success (CBOL Plant Working Group et al., 2009; Hollingsworth et al., 2011). The plastid coding region *matK* and the non-coding spacer *trnH-psbA* evolve more rapidly and generally provide a high level of species discrimination (Hilu and Liang, 1997; Kress et al., 2005; Lahaye et al., 2008; CBOL Plant Working Group et al., 2009; Pang et al., 2012). Similarly, the nuclear ITS region evolves quickly and has high discriminatory power, and has proven valuable for resolving relationships among closely related species (CBOL Plant Working Group, 2011; Xiong et al., 2022). The plastid *rps16*, often used as a supplementary DNA barcode exhibits high nucleotide variability polymorphism and evolutionary rates (Khan et al., 2013; Ryzhova et al., 2013; Fan et al., 2018; Li et al., 2025).

Cyperaceae, commonly known as the sedge family, comprises about 5687 species (Larridon, 2022), and ranks among the ten largest plant families, exhibiting a cosmopolitan distribution. However, its taxonomy poses significant challenges due to the highly reduced and complex floral characteristics of this family (Larridon et al., 2021). Diagnostic features in Cyperaceae, mostly lies in their fruits, which are not consistently available throughout the season (Dai et al., 2010; Simpson, 2019; Browning et al., 2020). Many species within Cyperaceae can propagate vegetatively through rhizomes, making them ecologically dominant in diverse ecosystems, including wetlands, river valleys, forest floors, and high-altitude and high-latitude herbaceous communities. The combination of taxonomic complexity, high species diversity, the absence of fruiting structures in certain seasons often makes conventional identification methods impractical. This is particularly evident in floristic and ecological studies, such as restoration ecology or animal diet analysis, where sedges are frequently identified only to genus level (e.g., *Carex* sp.) without resolving species-level identities (Hall and Zedler, 2010; Wang et al., 2017; Castle et al., 2020; Hartvig et al., 2021; Wang et al., 2024). As a result, sedges are often overlooked, not only by naturalists and amateurs but also by scientists (Starr et al., 2009).

While efforts have been initiated to assess the DNA barcoding potential of *matK*, *rbcL*, and ITS in the mega-diverse genus *Carex* (Starr et al., 2009; Clerc-Blain et al., 2010; Jiménez-Mejías et al., 2016), a family-wide DNA barcoding evaluation for Cyperaceae is still lacking. No standardized, empirically validated barcode recommendations exist for the family to date. In addition, pronounced taxonomic and geographic biases in existing reference datasets highlight the need to expand sampling across the family, particularly in underrepresented regions such as the Asian mountains. This situation emphasizes the critical need for robust molecular approaches, with DNA barcoding emerging as an effective tool for species identification in this globally important family.

Due to the limited variation observed in single-locus DNA barcodes for closely related taxa, DNA-barcoding approaches have emphasized the use of multi-locus markers. These resources have become more accessible and affordable with advancement in sequencing technologies (Robinson and Chalmers, 2012; Li et al., 2015). In this study, available gene sequences from GenBank were screened, and potential barcodes were selected based on their genetic diversity. Barcodes with high genetic diversity were further evaluated to test their discriminatory power within the family Cyperaceae. Finally, three plastid DNA (ptDNA) locus (*matK*, *rbcL, rps16*) and one nuclear ribosomal DNA (nrDNA) locus (ITS) were selected, and their universality and discriminatory power were assessed, both individually and in combination. This study marks the first global-scale attempt at DNA barcoding of the family Cyperaceae as a whole, utilizing all available DNA sequences in GenBank, alongside newly generated high-throughput sequencing data.

## 2 Materials and methods

### 2.1 GenBank sequences

All single-marker ptDNA sequences available for the family Cyperaceae were downloaded from GenBank. To ensure taxonomic accuracy, only sequences with voucher specimen information available and/or previously published were considered. The sequences with generic names only and shorter than 300 base pairs were excluded to enhance data reliability.

Sequences were aligned using MAFFT v7.526 (Nakamura et al., 2018) with default settings and manually adjusted with BIOEDIT v7.0.5.3 (Hall, 1999) with emphasis on gap-flanking regions and matrix ends. Unaligned sequences were removed or trimmed. The cleaned sequences underwent screening with DNAsp v6.0 (Rozas et al., 2017), focusing on nucleotide diversity (π). Markers with high values were selected except for those representing very few genera or with a low number of sequences in GenBank. All available sequences from ITS for the family Cyperaceae were also downloaded, aligned, and manually edited.

### 2.2 New sequences

#### 2.2.1 Sampling

A total of 603 samples representing 164 Cyperaceae species were collected from the Hengduan Mountains (China, North Myanmar) and the Himalayas (Nepal). These areas are one of the main gaps in the sequencing of Cyperaceae (Roalson et al., 2021), despite also constituting a hotspot of species diversity (Myers et al., 2000; Wambulwa et al., 2021). Whenever possible, for each species, several collections were made from different populations. The identification of these vouchers was verified by examining the morphological characteristics, consulting national and regional Floras and Monographs (Clarke, 1893; Kükenthal, 1909, 1936; Kern, 1974; Koyama, 1978, 1985; Rao and Verma, 1982; Simpson and Koyama, 1998; Kukkonen, 2001; Dai et al., 2010; Dey and Prasanna, 2015; Simpson, 2019; Jin and Zhang, 2020; Shrestha et al., 2022), as well as by comparing the specimens with the respective types.

#### 2.2.2 Primer design

Primer pairs were designed for the three ptDNA regions (*matK*, *rbcL*, and *rps16*). The primer design process involved downloading the target gene sequence from GenBank, followed by sequence alignment using MAFFT v7.526 (Nakamura et al., 2018). The aligned sequences were then imported into Sequencer v5.4.6, which allowed identification of conserved regions within the consensus sequences. These conserved regions were selected as the primer pairs (**Table 1**).

**Table 1:** PCR primers used on the first-round amplification. Primer pairs for ITS1 and ITS2 were sourced from Cheng et al. (2016). All the other primers are newly designed in this study.

Barcode	Primer name	Primer sequence (5’-3’)
ITS1	ITS-P5	CCTTATCAYTTAGAGGAAGGAG
	ITS-U2	GCGTTCAAAGAYTCGATGRTTC
ITS2	ITS-P3	YGACTCTCGGCAACGGATA
	ITS-U4	RGTTTCTTTTCCTCCGCTTA
*matK*1	matK_F1	GCTATGAYAWTAAATCTAG
	matK_R1	GGAYCCAGCATTGAAGGATTTG
*matK*2	matK_F2	CCTTATCCTRTCCATTTTGAAATC
	matK_R2	GAATWGCTTTRCCTTGATATCG
*matK3*	matK_F3	GGWTCTTCACAGATCCTCTCATG
	matK_R3	GTCCARATYGGYTTACTAGTAG
*matK*4	matK_F4	CACTTTGTACTGTAKTRGGACATC
	matK_R4	GATCATKAATAYGRGTAATATC
*rbcL*1	rbcL_F1	GGATTTAAAGCAGGGGTTAAAG
	rbcL_R1	CCATGAGGTGGRCCTTGGAAAG
*rbcL*2	rbcL_F2	CTATTGTAGGTAATGTATTTGG
	rbcL_R2	CATTTCTTCASATGTASCTGCAG
*rbcL*3	rbcL_F3	CCATTTATGCGTTGGAGAGAYCG
	rbcL_R3	CACGTARTAARTCAACAAAACC
*rbcL4*	rbcL_F4	GAGATCATATTCACKCTGGTACAG
	rbcL_R4	GATTGCTTTCCATACTTCACAAGC
*rps16*_1	rps16_F1	GGTTCGACATAATTTGTTCTG
	rps16_R1	CTTTATTYCTTTCAAARGGAAAGC
*rps16*_2	rps16_F2	GGTGGAAKGGRARTGAATYC
	rps16_R2	GACTATGATYCAAATAGARTTC

The newly designed primer met the following criteria: (1) Primer length of 18–24 bp (normally 20 bp); (2) amplified fragment length of 350–430 bp; (3) no mismatch from the first 5–6 bases of the 3’ end; (4) 40-60% G/C content; (5) start and end with 1–2 G/C pairs; (6) no complementary regions between primer pairs; and (7) forward and reverse primers oriented in opposite directions. The ptDNA region *rbcL* and *matK* were divided into four parts, and the *rps16* was divided into two parts, ranging from 360 to 430 bp in length.

#### 2.2.3 DNA extraction, amplification, and sequencing

Total genomic DNA was isolated from silica-dried leaf and culm material using the modified CTAB method (Li et al., 2013), following the manufacturer’s protocols. Gel electrophoresis on a 1% agarose gel was employed to assess DNA quality based on fragment length. Subsequently, poor-quality and colored DNA products were purified using a Universal DNA Purification and Recovery Kit (Cat: DC013-100, GeneBetter, China).

Newly designed primers were used for the ptDNA regions (*matK*, *rbcL*, and *rps16*), while for ITS, we used the universal primers reported by Cheng et al. (2016) (**Table 1**; **Supplementary Figures 1**–**4**). A two-round amplification procedure was implemented to accommodate the high-throughput sequencing method. In the first round of PCR, a 10 μL volume contained 1μL genomic DNA, 5μL master mix, 3μL double-distilled water (dd water), and 1 μL 5μM primer pairs. The amplification steps for the first round included an initial cycle at 95 °C for 2 min, followed by 40 cycles at 95 °C for 15 s, 52 °C for 15 s, 72 °C for 10 s, and one cycle at 72 °C for 1 min. The amplifying primers utilized in the first round were synthesized with a fixed 14 bp linker sequence (GTAGACTGCGTACC) at the 5’-end (Xu and Hong, 2021). The second round of PCR was conducted in a 10 μL volume, consisting of 1μL product from the 1st round of PCR, 5μL master mix, 3μL dd water, and 1 μL barcode primers. The amplification steps for the second round were as follows: an initial cycle at 95 °C for 1 min, followed by 35 cycles at 95 °C for 15 s, 55 °C for 15 s, 72 °C for 10 s; and one cycle at 72 °C for 1 min. Notably, a sample-specific barcoded primer was used in the second round of PCR, allowing the resulting PCR amplicons to be tagged with a unique 10 bp barcode. Amplicons with different barcodes were mixed to form an amplicon pool and purified to construct a sequencing library with the NEXTflex Rapid DNA-Seq Kit (#5144-01), following the manufacturer’s protocol. Subsequently, the libraries were quantified before sequencing on the Illumina HiSeq 2500 platform using the PE250 mode at Sangon Biotech Company (Shanghai) (Xu and Hong, 2021).

#### 2.2.4 Sequenced data preprocessing

Paired-end reads from Illumina HiSeq 2500 were merged using FLASH v1.2.11 (Magoč and Salzberg, 2011) with default settings, and uncombined reads were excluded. Subsequently, reads were trimmed at the 3’-end using a custom Python script. The trimmed reads were processed with the ‘divide’ script (version 4.1, https://github.com/wpwupingwp/divide), grouping reads to distinct genomic fragments by recognizing the barcode primer sequences. Consensus sequences for each fragment per sample were generated using Vsearch available at https://github.com/torognes/vsearchun (Rognes et al., 2016). A 0.9 sequence similarity threshold was applied, and the consensus with the highest sequencing depth was selected as the final sequence for each sample (Xu and Hong, 2021). Subsequently, fragments from each DNA region were sequentially joined using a ‘concatenate’ function in Phylosuite v1.2.3 (Zhang et al., 2020).

### 2.3 Data integration and alignment

Sequences retrieved from GenBank and those newly sequenced were combined into FASTA files. In total, 6739 sequences representing 1278 species were used in the data analysis, covering the four selected markers. Only species represented by two or more accessions (sequences) were included in the analysis. Species represented by a single sequence are not informative for DNA barcoding, which requires multiple accessions to assess species boundaries and genetic variation accurately.

The sequences were then aligned with MAFFT v7.526 (Nakamura et al., 2018), and manual adjustments to the alignments were made using BIOEDIT 7.0.5.3 (Hall, 1999). Unaligned or unreliable sequences or regions of sequences were removed or trimmed before proceeding to downstream analyses.

### 2.4 Locus performance

The base composition, genetic distances, variable sites, conserved sites, and parsimony-informative site values were analyzed using MEGA v6.06, following the Kimura-2-parameter (K2P) model (Kimura, 1980). To assess individual and combined locus performance, three different analytical approaches were employed: (1) barcode gap; (2) sequence similarity; and (3) phylogenetic placement.

#### 2.4.1 Barcode gap

Utilizing the Kimura-2-parameter model in MEGA v6.06 (Tamura et al., 2013), intraspecific and interspecific distances were calculated. For each sample, maximum intraspecific distances and minimum interspecific distances relative to others were computed. Barcode gaps were identified when the minimum interspecific distance exceeded the maximum intraspecific distance (Meyer and Paulay, 2005). Visual diagrams of barcode gaps were created as scatter plots using the ggplot2 package in R v4.3.1 (Wickham, 2016; R Core Team, 2023). The percentage of species with barcode gaps for each marker was then calculated.

#### 2.4.2 Sequence similarity

Best match (BM) and best close match (BCM) functions in TaxonDNA v1.10 (Meier et al., 2006) were used to evaluate the identification success of individual barcodes and combinations. BM analysis aimed to find the closest DNA barcode match for a given query. If the closest match corresponds to the same species as the query, the identification was considered correct, incorrect when they belonged to different species, or ambiguous if the matches are from both the same and different species (Meier et al., 2006; Raskoti and Ale, 2021). In BCM analysis, a 95% genetic distance threshold was applied based on the intraspecific distances. Queries without any barcode matches below this threshold were treated as unidentified. For the remaining queries, identification was considered correct if the closest match within the threshold was the same species, a failure if it was a different species, and ambiguous if multiple equally good matches were found involving at least two species (Meier et al., 2006).

#### 2.4.3 Phylogenetic placement

**Figure 1:**
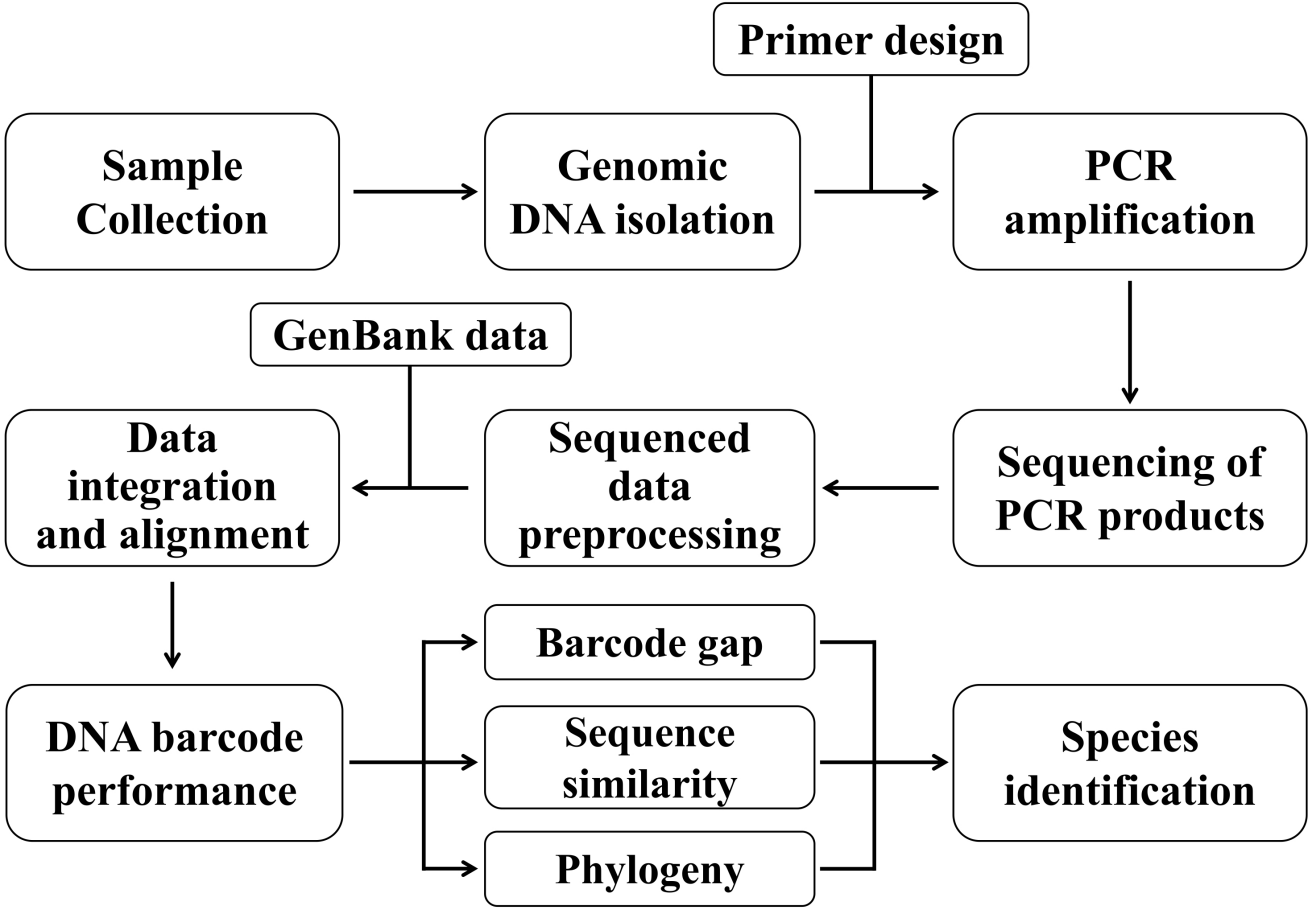
Schematic diagram of DNA barcoding workflow.

Maximum Likelihood (ML) trees were constructed using the RAxML v8.2.X program (Stamatakis, 2014) under the GTRGAMMA model, with 1000 bootstrap replicates. A species was considered successfully identified when all the samples of that species formed a monophyletic clade, with a node support value >50. The proportion of monophyletic species was converted into a percentage. The overall workflow of the present study is provided in **Figure 1**.

## 3 Results

### 3.1 Selected DNA regions

Sixteen ptDNA locus were initially selected based on their polymorphism as indicated by DNAsp (**Table 2**). However, considering the availability of these regions for various species in GenBank, we focused on three ptDNA locus representing the highest number of species, along with their polymorphism: *matK*, *rbcL*, *rps16*. Additionally, ITS was added.

**Table 2:** Sequence characteristics of coding and non-coding genes.

Genes	Genera	Species	Sequences	Aligned sequence	S	Eta	Hap	Pi
*atpB_rbcL*	1	84	204	554	84	90	54	0.018
*atpF_atpH*	3	63	121	477.2	19.8	21.8	8.9	0.025
*atpI_atpH*	1	44	114	750	99	112	47	0.017
*matK*	30	1131	6026	768.7	41.4	45.8	14.5	0.013
*ndhF*	39	508	778	1268.7	57.9	65.6	11.9	0.015
*petN_psbM*	2	77	269	380	40	44.5	30	0.016
*psbA_trnH*	20	123	729	763.1	68.2	73.7	12	0.063
*psbN_psbB*	14	84	88	619.6	23.6	25	5.71	0.016
*rbcL*	71	1122	4185	1006	23.4	24.3	7.3	0.015
*rpl16*	1	62	175	733	125	142	105	0.02
*rpoB*	3	80	195	724.3	22.3	24.3	15.7	0.007
*rps16*	36	739	1826	769.1	40	46.7	18.1	0.016
*trnC_ycf6*	2	110	189	479.5	83	103	58	0.026
*trnK*	1	107	616	594	102	130	96	0.017
*trnL-F*	56	836	1304	827.6	46.8	51.7	11.4	0.027
*ycf6_psbM*	2	200	395	352.5	56.5	68.5	46	0.013

S, number of polymorphic sites; Eta, total number of mutations; Hap, number of haplotypes; Pi, nucleotide diversity.

A total of 4948 sequences representing 1230 species were retrieved from GenBank, while 1791 sequences from 164 species were newly generated using Illumina technology. The newly generated ITS sequences were deposited in GenBank (NCBI), whereas the plastid DNA (ptDNA) sequences were deposited in GenBase; all accession numbers are provided in **Supplementary Table S1**. Altogether, the final dataset comprised 6739 sequences representing 1278 species (**Supplementary Table 1**), accounting for 58% of the genera, and 22% of the global Cyperaceae species. All 24 tribes of Cyperaceae are represented in this study. However, sequencing progress across the tribes is uneven, leading to inadequate sampling for many tribes.

ITS was represented by 3654 sequences, *matK* by 560 sequences, *rbcL* by 851 sequences, and *rps16* by 1678 sequences. The sizes of aligned sequences varied across markers, ranging from 334 bp to 678 bp for ITS, 959 bp to 1336 bp for *matK*, 995 bp to 1316 bp for *rbcL*, and 306 bp to 898 bp for *rps16*.

### 3.2 Locus performance

#### 3.2.1 Genetic distances and barcode gaps

Among the single-locus barcodes, the pairwise intraspecific distances varied between 0 and 0.17, and the interspecific distance varied between 0 and 0.22. ITS showed the highest interspecific variation (0 – 0.22), and *rbcL* showed the least interspecific variation (0 – 0.04). Similarly, *rps16* showed the highest intraspecific variation (0 – 0.17), while *matK* was relatively conserved, ranging from 0 to 0.09. Among the multi-locus combinations, *matK* + *rps16* showed the highest intraspecific variation (0 – 0.10), and *rbcL* + ITS with the least intraspecific variation (0 – 0.03). Similarly, *rps16* + ITS showed the highest interspecific variation (0 – 0.19), and *rbcL* + *rps16* with the least interspecific variation (0 – 0.07).

**Figure 2:**
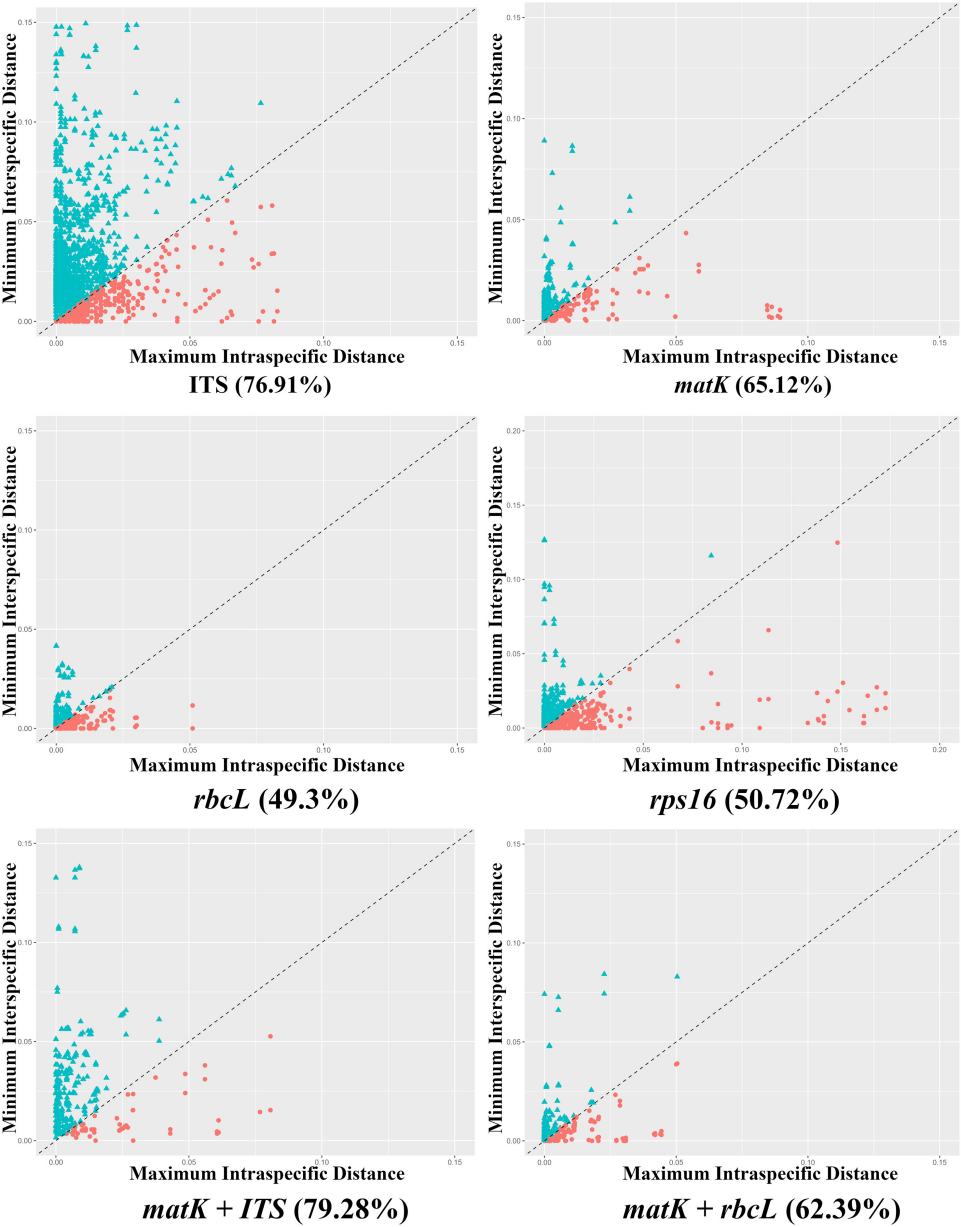
Genetic distance and barcode gaps for single-locus and their combinations. The x-axis denotes the maximum intraspecific distance and the y-axis denotes the minimum interspecific distances. Dots above the 1:1 slope line indicate the barcode gaps.

**Figure 3:**
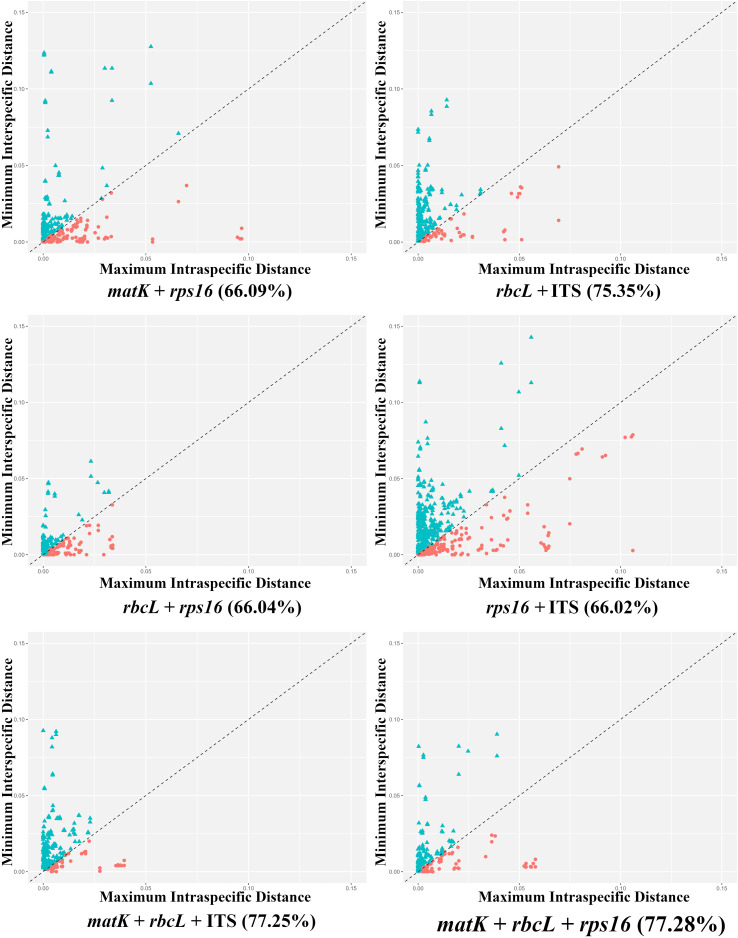
Genetic distance and barcode gaps for multi-locus combinations.

**Figure 4:**
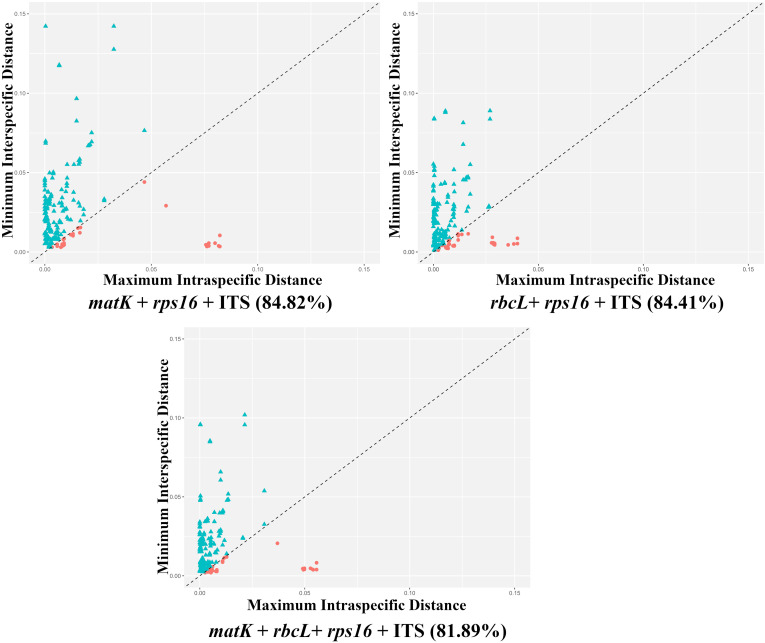
Genetic distance and barcode gaps for multi-locus combinations.

Barcode gaps, based on scatter plots showed different resolutions between single-locus and multi-locus barcodes. Among single loci, ITS showed the highest barcode gaps (76.91%), followed by *matK* (65.12%), *rps16* (50.72%), and *rbcL* (49.30%). The highest barcode gaps for combination of two-locus was observed for *matK* + ITS with 79.28% success rate. The lowest barcode gaps were shown by *matK* + *rbcL* (62.39%). The combination of three-locus, *matK* + *rps16* + ITS and *rbcL* + *rps16* + ITS demonstrates nearly equal barcode gaps, i.e. 84.82% and 84.41%, respectively. The four-locus combination, however, showed 81.89% barcode gaps (**Table 3**, **Figures 2**–**4**).

**Table 3:** Sequence characteristics and species identification success rates.

Gene	Species	No. of seq.	No. of bases (mean)	MaxIntra_dis (range)	MaxIntra_dis (mean)	MinInter_dis (range)	MinInter_dis (mean)	BM %	BCM %	Barcode gaps %	Phylogeny (ML)
*ITS*	1154	3654	605	0–0.08	0.006	0–0.22	0.02	87.3	86.5	76.91	88.7
*matK*	152	560	1314	0–0.09	0.007	0–0.09	0.009	86.8	86.3	65.12	82.2
*rps16*	443	1678	735	0–0.17	0.008	0–0.13	0.007	62.9	62.4	50.72	77.3
*rbcL*	240	851	1307	0–0.05	0.003	0–0.04	0.003	77.9	77.8	49.3	69.6
*matK + rbcL*	132	476	2664	0–0.05	0.005	0–0.084	0.007	80.3	79	62.39	82.4
*matK + rps16*	141	522	2032	0–0.97	0.006	0.0–128	0.011	75.9	75.9	66.09	83.1
*matK + ITS*	115	362	1939	0–0.81	0.008	0–0.138	0.024	88.4	88.4	79.28	90
*rbcL + rps16*	133	480	2006	0–0.034	0.004	0–0.061	0.006	74.4	74.4	65.02	82.5
*rbcL + ITS*	109	353	1929	0–0.069	0.007	0–0.093	0.018	88.4	87.5	75.35	85
*rps16 + ITS*	223	869	2149	0–0.106	0.009	0–0.175	0.015	76.2	76.2	66.04	88.2
*matK + rbcL + rps16*	126	467	3358	0–0.06	0.005	0–0.09	0.009	73.7	73.7	77.28	86.3
*matK + rbcL + ITS*	76	254	3323	0–0.04	0.005	0–0.09	0.014	89.8	89	77.75	92.9
*matK + rps16 + ITS*	81	263	2669	0–0.08	0.009	0–0.14	0.026	85.2	84.8	84.82	95.4
*rbcL + rps16 + ITS*	82	267	2636	0–0.04	0.006	0–0.09	0.02	84.6	84.6	84.41	93.8
*matK + rbcL + rps16 + ITS*	77	248	3977	0–0.06	0.006	0–0.10	0.018	83.9	83.5	81.89	93.7

#### 3.2.2 Sequence similarity

**Figure 5:**
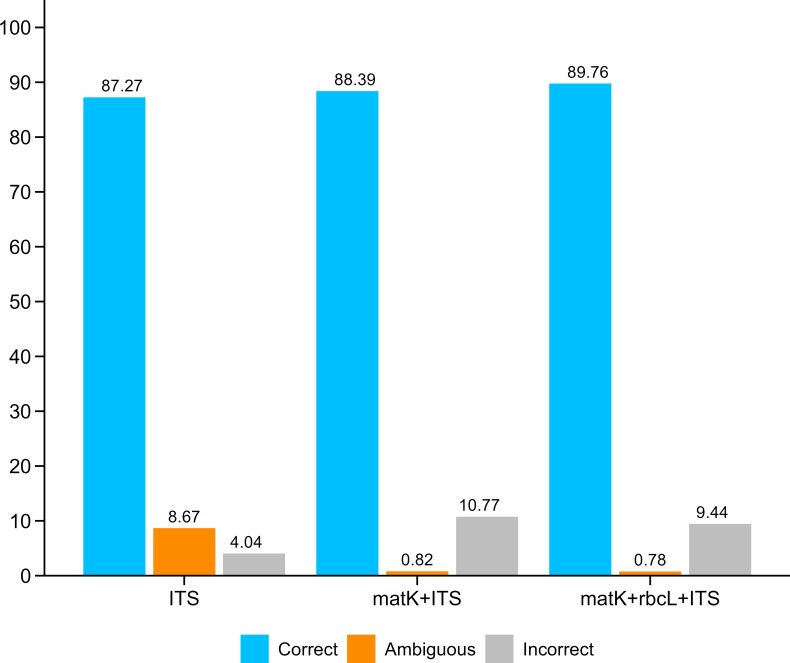
Best match (BM) analysis for the candidate barcodes for Cyperaceae.

**Figure 6:**
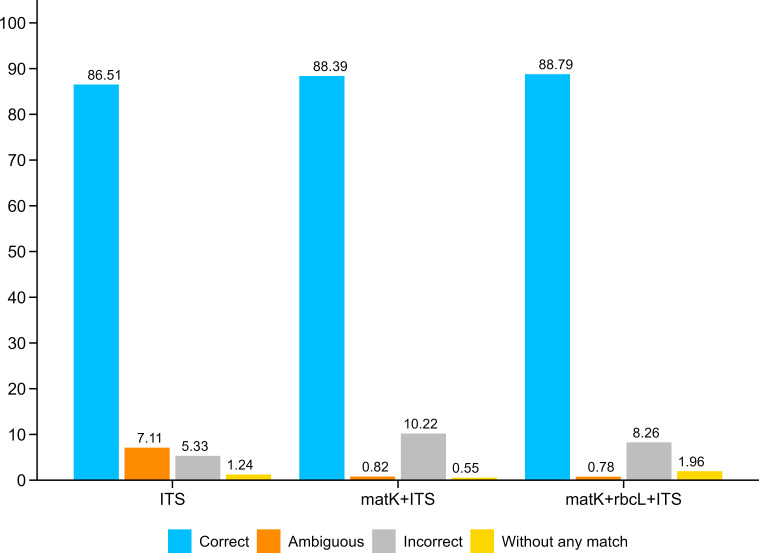
Best close match (BCM) analysis for the candidate barcodes for Cyperaceae.

The best match and best close match analysis demonstrated a distinct resolution among the single-locus and multi-locus barcodes. Among the single-locus, ITS showed the highest success in species discrimination (BM: 87.27% and BCM: 86.51%), followed by *matK* (BM and BCM: 86%), *rps16* (BM and BCM: 77%), and *rbcL* yielded poor results (BM and BCM: 62%). Among the multi-locus combinations, *matK* + ITS yielded the best results (BM and BCM: 88.39%), followed by *rbcL* + ITS (BM and BCM: 88.38% and 87.53%), *matK* + *rbcL* (BM and BCM: 80.25% and 78.99%), and *rps16* + ITS (BM and BCM: 76.17%). For three-locus combinations, *matK* + *rbcL* + ITS proved to be the best locus with BM and BCM, 89.76% and 88.97%, respectively (**Figures 5**, **6**).

#### 3.2.3 Phylogenetic placement

**Figure 7:**
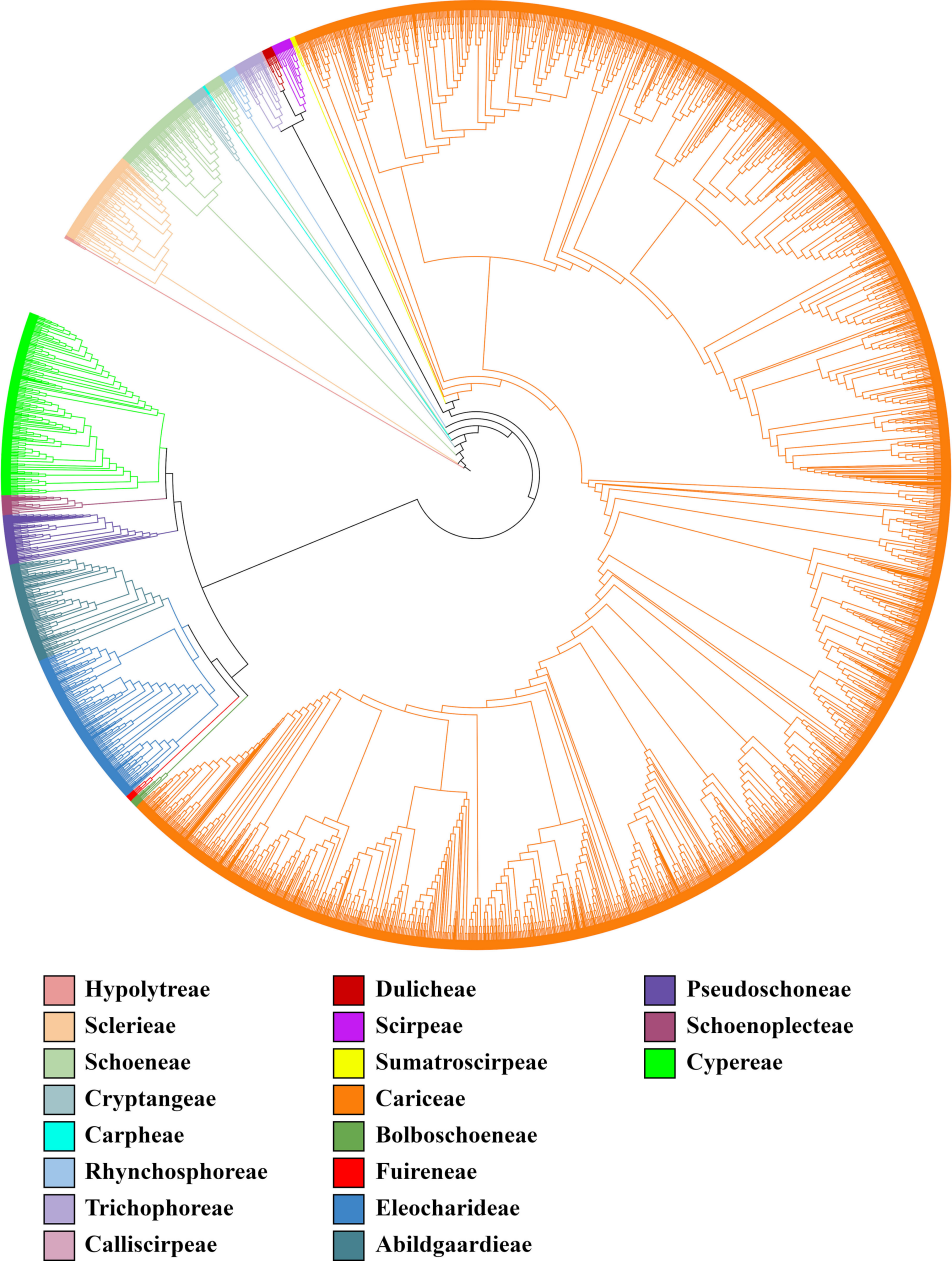
Maximum likelihood tree based on the ITS barcode.

A phylogenetic tree was constructed for each candidate barcode using the maximum likelihood (ML) method to check the species discriminatory power, thereby evaluating the monophyly rate. Species discrimination rates varied among single-locus ranging from 69.6% (*rbcL*) to 88.7% (ITS), where *matK* performed 82.2%, and *rps16* performed 77.3%, respectively. Among the two-locus combinations, discrimination rates ranged from 82.4% (*matK* + *rbcL*) to 90% (*matK* + ITS). In the three-locus combinations, the highest species discrimination was observed for *matK* + *rps16* + ITS, reaching 95.4% (**Table 3**). Overall, the phylogenetic tree method identified ITS as the most effective barcode for species discrimination of Cyperaceae species (**Figure 7**).

### 3.3 Taxonomic and geographic representation of Cyperaceae in molecular datasets

**Figure 8:**
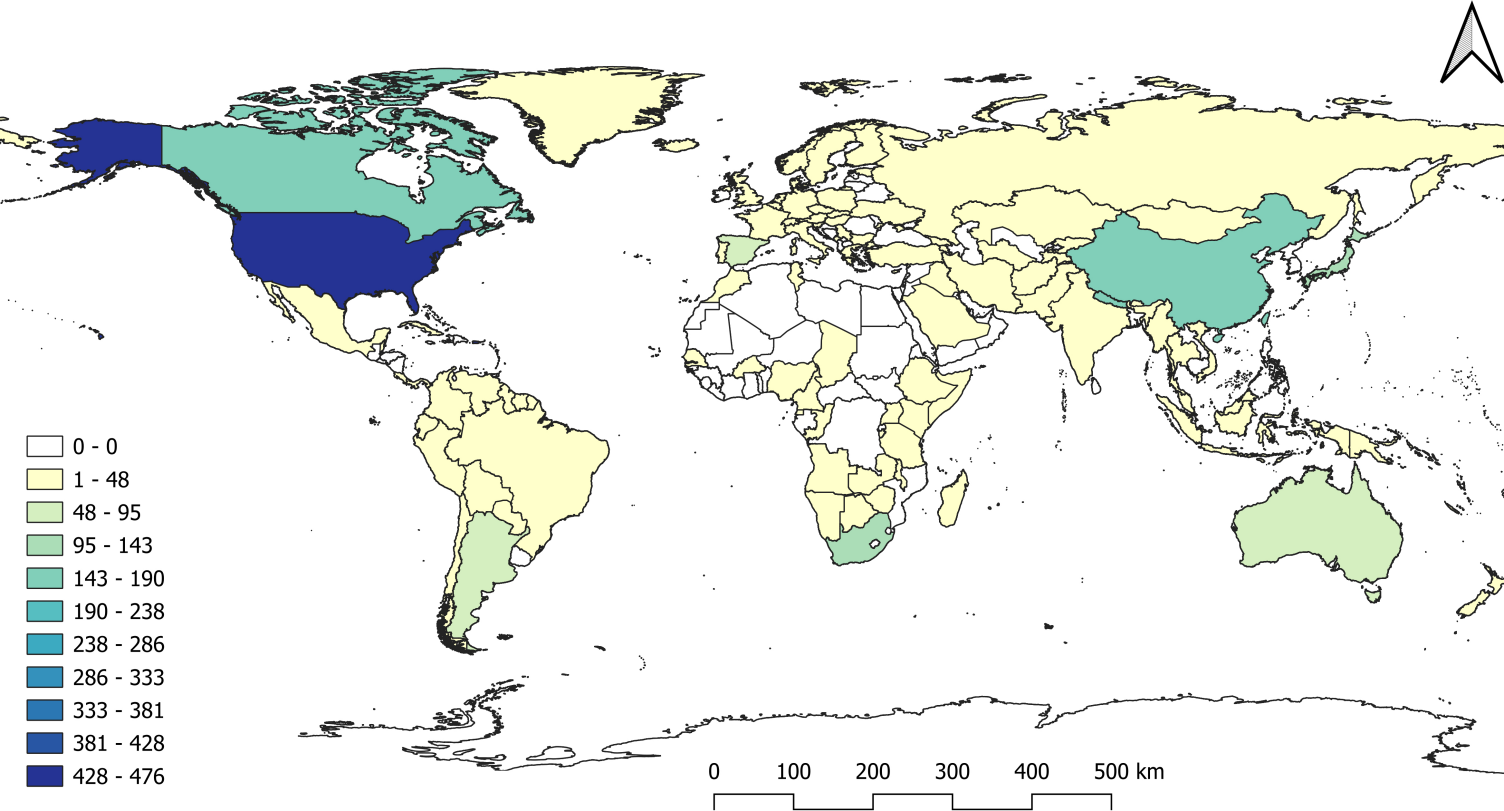
Geographic distribution of published Cyperaceae DNA sequences.

The species-rich tribe Cariceae, which includes the mega-diverse genus *Carex*, is partially represented, with 41% of its species sequenced. In contrast, other species-rich tribes: Cypereae, Abildgaardieae, Schoeneae, Rhynchosporeae, Sclerieae, Eleocharideae, and Hypolytreae are represented by 10%, 8%, 15%, 4%, 18%, 23%, and 1% of their species, respectively. Similarly, moderately species-rich tribes such as Scirpeae, Pseudoschoeneae, and Cryptangieae are represented by 27%, 23%, and 22% of their species, respectively (**Table 4**). Geographically, north America is relatively well sampled, whereas south Asia, southeast Asia and tropical Africa are poorly sampled (**Figure 8**).

**Table 4:** Cyperaceae tribes and the sequence representation in this study.

Tribe	Total number of species (Larridon, 2022)	Number of species sequenced	% of species sequenced
Abildgaardieae	571	43	8
Bisboeckelereae	24	1	4
Bolboschoeneae	15	3	20
Calliscirpeae	2	1	50
Cariceae	2003	830	41
Carpheae	18	2	11
Chrysitricheae	14	1	7
Cladieae	3	2	67
Cryptangieae	50	11	22
Cypereae	1130	115	10
Dulicheae	5	2	40
Eleocharideae	302	70	23
Fuireneae	55	4	7
Hypolytreae	172	2	1
Khaosokieae	1	1	100
Pseudoschoeneae	64	15	23
Rhynchosporeae	399	14	4
Schoeneae	466	70	15
Schoenoplecteae	17	10	59
Scirpeae	73	20	27
Sclerieae	258	46	18
Sumatroscirpeae	4	1	25
Trichophoreae	19	13	68
Trilepideae	16	1	6

## 4 Discussion

This study utilized GenBank and freshly sequenced data to analyze barcoding potential of three ptDNA (*matK, rbcL*, *rps16*) and one nrDNA (ITS) and their combination. ITS showed the highest interspecific divergence, low intraspecific variation, a strong barcode gap, and high species discrimination success among the single-locus markers. This rapidly evolving nuclear gene, known for its high variability, is also a well-supported universal plant barcode (Kress et al., 2005; CBOL Plant Working Group, 2011; Raskoti and Ale, 2021; Rather et al., 2023). However, the utility of ITS is often constrained by genomic complications, including paralogy, pseudogene formation, and incomplete concerted evolution (King and Roalson, 2008; Hollingsworth et al., 2009; Gehrke et al., 2010; CBOL Plant Working Group, 2011; Hochbach et al., 2018).

The plastid region *matK* was the second most effective marker for species delimitation, and is supported as a core region for plant barcoding by multiple studies (Kress et al., 2005; CBOL Plant Working Group et al., 2009; Hollingsworth et al., 2009; Chen et al., 2010; Rather et al., 2023). Because short *matK* fragments provided low species resolution, recovering an informative 1300 bp region required four primer pairs for high-throughput sequencing, highlighting a practical limitation of this marker. When *matK* cannot be fully amplified, *rps16* can serve as an alternative marker, complementing ITS.

In contrast, the ptDNA *rbcL*, often regarded as a universal barcode (Hasebe et al., 1995; Hollingsworth et al., 2009; Li et al., 2012), was found to have minimal interspecific variation and low resolution in phylogenetic trees, indicating its poor effectiveness for distinguishing Cyperaceae species. This observation aligns with findings from other plant groups, where the conservative evolution of *rbcL* limits its utility in discriminating closely related species (Kress et al., 2005; Li et al., 2012).

Single DNA barcode regions often lack sufficient genetic variation for effective barcoding (CBOL Plant Working Group, 2011; Rather et al., 2023). In contrast, multi-locus barcodes demonstrated higher species discrimination compared to single‐locus barcodes (CBOL Plant Working Group, 2011; Yan et al., 2015b; Rather et al., 2023). In this study, the ITS + *matK* combination achieved the highest species discrimination, consistent with previous studies (Li et al., 2016; Raskoti and Ale, 2021; Wang et al., 2022). The alternative combination of ITS + *rps16* is suggested as a supplementary choice, especially when *matK* amplification is unsuccessful (Chen et al., 2015; Raskoti and Ale, 2021). Other two-locus plastid combinations (*matK* + *rbcL*, *matK* + *rps16*, *rbcL* + *rps16*) showed limited effectiveness, likely due to the conserved nature of these markers and their insufficient variable sites (CBOL Plant Working Group, 2011; Raskoti and Ale, 2021).

Resolution rates improved significantly with the use of a three-locus combination, particularly *matK* + *rps16* + ITS. This multi-locus dataset showed 84.8% barcode gaps, 85.2% BM, 84.8% BCM, and a species identification success of 95.4%, representing the highest discrimination among both single-locus and multi-locus barcodes tested. The effectiveness of this combination likely reflects complementary sequence variation across nuclear and plastid genomes (Fazekas et al., 2008; Daru et al., 2017). The rapid evolution of ITS, together with the universality of *matK* and the high polymorphism of *rps16*, is likely to have a synergistic effect that enhance taxonomic resolution in complex family such as Cyperaceae (CBOL Plant Working Group et al., 2009, 2011; Fan et al., 2018; Li et al., 2025).

Developing a comprehensive reference gene sequence is crucial for advancing our understanding of taxonomy, phylogeny and evolution of Cyperaceae. However, two major gaps remain: geographic and taxonomic. Although Cyperaceae is dominant in different ecosystems worldwide, sampling efforts are unevenly distributed, with species-rich regions such as southeast Asia, the Himalayas, and tropical Africa being particularly under-sampled.

Moreover, DNA sequence coverage across the family is inconsistent, especially in species-rich tribes such as Cariceae, Cypereae, Schoeneae, Rhynchosporeae, Sclerieae, and Hypolytreae. For example, although the mega diverse genus *Carex* appears relatively well sampled, more than 60% of its (> 1000 species) have yet to be sequenced. Similarly, the species-rich genus *Cyperus* still lacks DNA sequence data for more than 80% of its species. Such uneven geographic and taxonomic sampling may influence estimates of barcode performance. Furthermore, barcode performance may vary among tribes due to differences in evolutionary rate, genomic structure, and hybridization patterns across the family. Further lineage focused barcoding studies, together with expanded sampling across under-sampled tribes and regions, will help refine barcode recommendations and ensure adequate representation of Cyperaceae diversity.

## 5 Conclusion

This study presents a comprehensive analysis of DNA barcodes for the family Cyperaceae, using four DNA regions: *matK*, *rbcL*, *rps16*, and ITS. The results revealed that the nuclear ITS region and the plastid *matK* region are effective in accurately identifying the majority of Cyperaceae species. Furthermore, *rps16* emerged as a potential supplementary barcode for this family. Given the intricate taxonomy of Cyperaceae, a combination of DNA markers, specifically *matK* and ITS, is recommended for species identification. Relying solely on plastid DNA markers could lead to misidentifications. Notably, incorporating the ITS marker alongside plastid markers (*matK* and either *rbcL* or *rps16*) greatly enhances the accuracy of species identification. These findings provide valuable insights to guide future efforts for the precise identification of Cyperaceae species.

A total of 6739 sequences representing 1278 Cyperaceae species (58% of the total genera, and 22% of the family’s total species) were analyzed in this study. Among these, 1791 sequences from 164 species were newly generated. All 24 tribes of Cyperaceae were represented; however, sampling coverage varied considerably among tribes. For example, the species-rich tribes, Cariceae and Cypereae, had approximately 40% and 10% of its species sequenced, respectively, to date. Species-poor and monotypic genera were generally well represented, though some remain under-sampled due to limited available material or taxonomic uncertainties.

Geographically, significant gaps remain, particularly in regions such as tropical Africa, Southeast Asia, and South America, which are known as the center of diversity of Cyperaceae. Addressing these gaps through intensified and targeted sampling is urgently needed, especially in species-rich tribes such as Cariceae, Cypereae, Schoeneae, Rhynchosporeae, Sclerieae, and Hypolytreae. Expanding these efforts is crucial for building a robust and comprehensive reference GenBank database for the family Cyperaceae, which will facilitate future taxonomic, ecological, and conservation research.

## Data Availability

The ITS sequences generated in this study have been deposited in GenBank (NCBI) and are publicly available at https://www.ncbi.nlm.nih.gov/nucleotide/, with accession numbers listed in **Supplementary Table 1**. The pladtid DNA sequences have been deposited in GenBank in the National Genomics Data Center, Beijing Institute of Genomics, Chinese Academy of Sciences/China National Center for Bioinformation, under accession numbers C_AA477148.1 to C_AA478650.1 and are publicly accessible at https://ngdc.cncb.ac.cn/genbase.
