# Supplementary material for: A universal DNA-barcode approach to aid species identification in the family Cyperaceae
Source: Front Plant Sci. 2026 Jul 16;17:1794327. doi: 10.3389/fpls.2026.1794327 (PMC13421178; doi:10.3389/fpls.2026.1794327)
Supplement: Supplementary file 1 [file DataSheet1.docx]

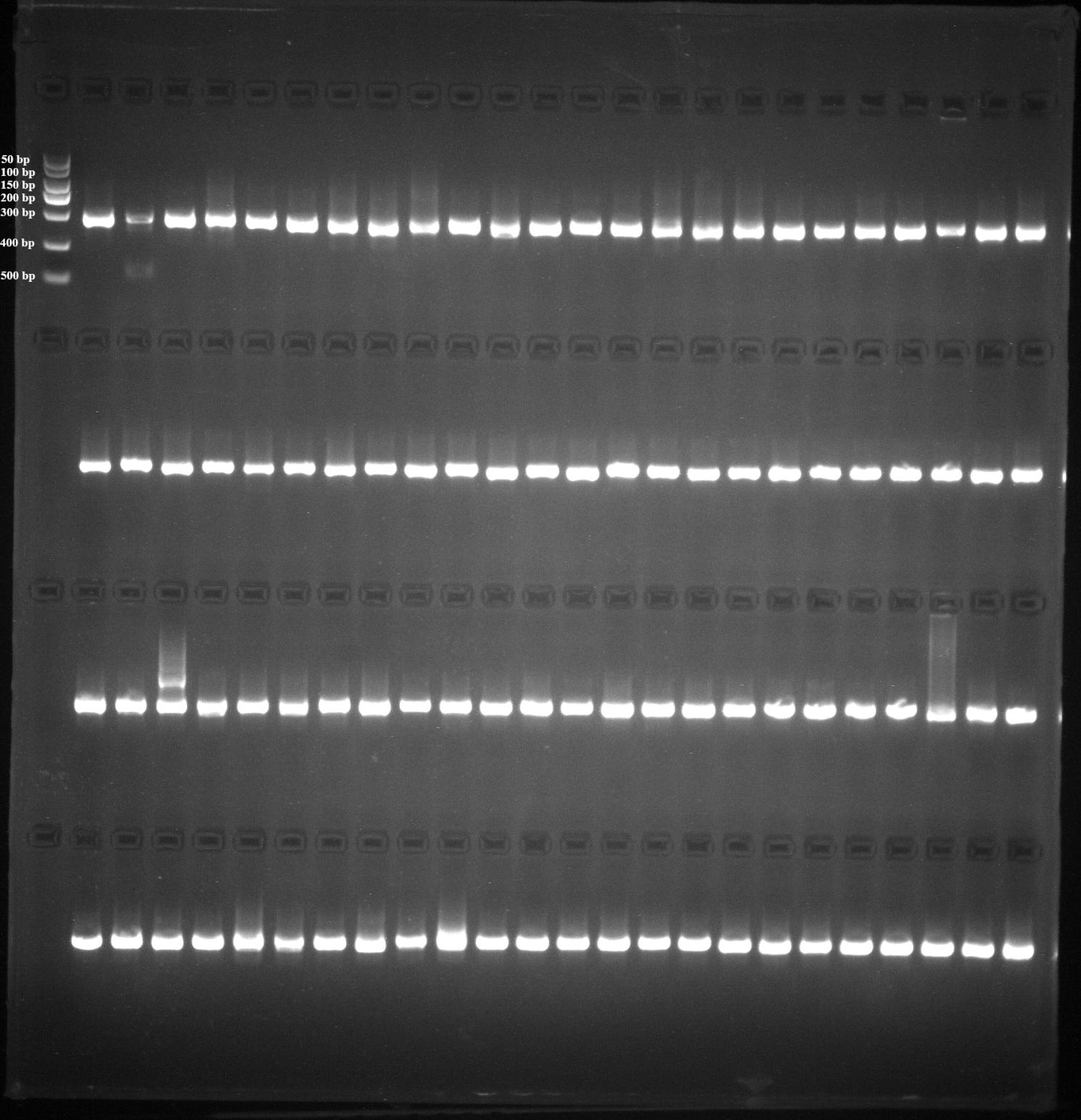


Supplementary Fig. 1. Agarose gel electrophoresis of PCR-amplified ITS region.


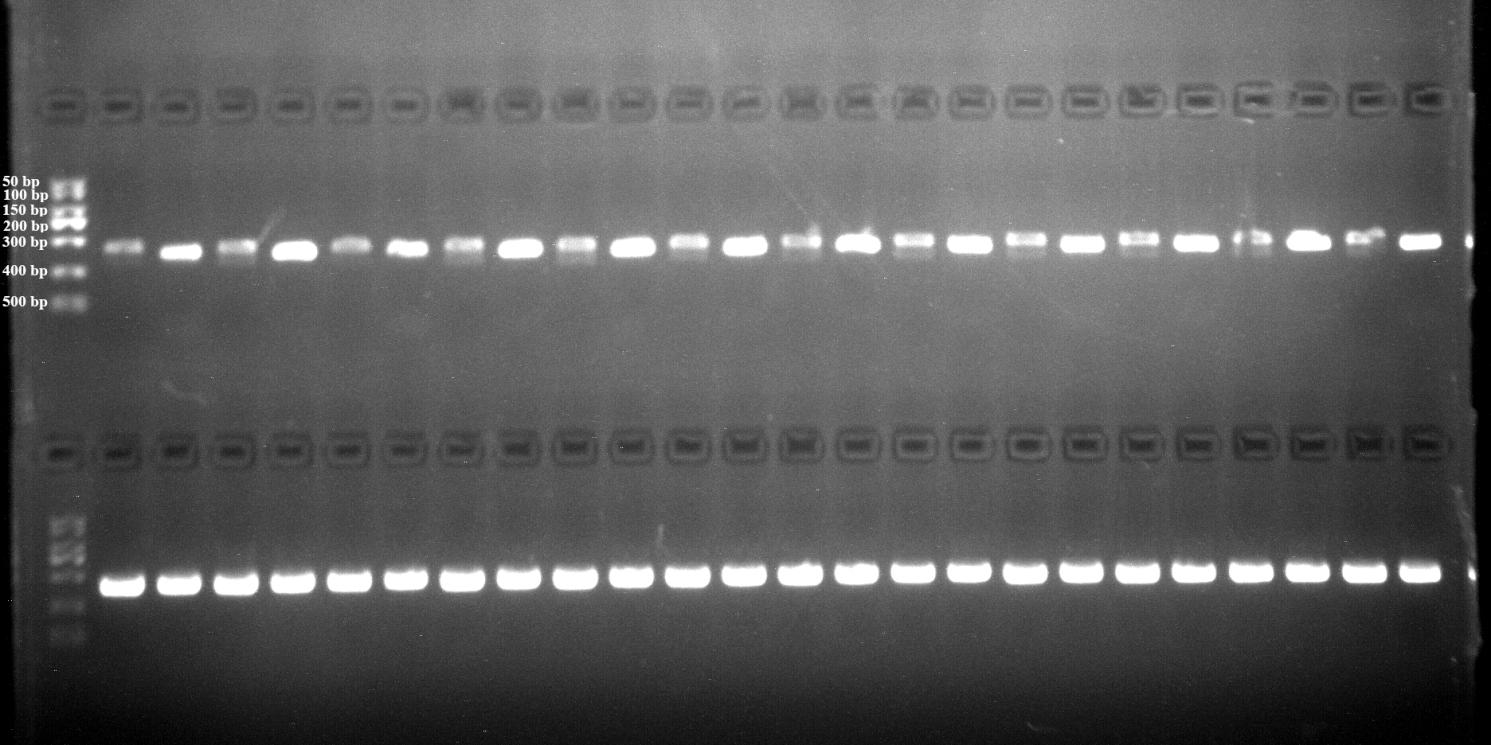


Supplementary Fig. 2. Agarose gel electrophoresis of PCR-amplified *matK* region.


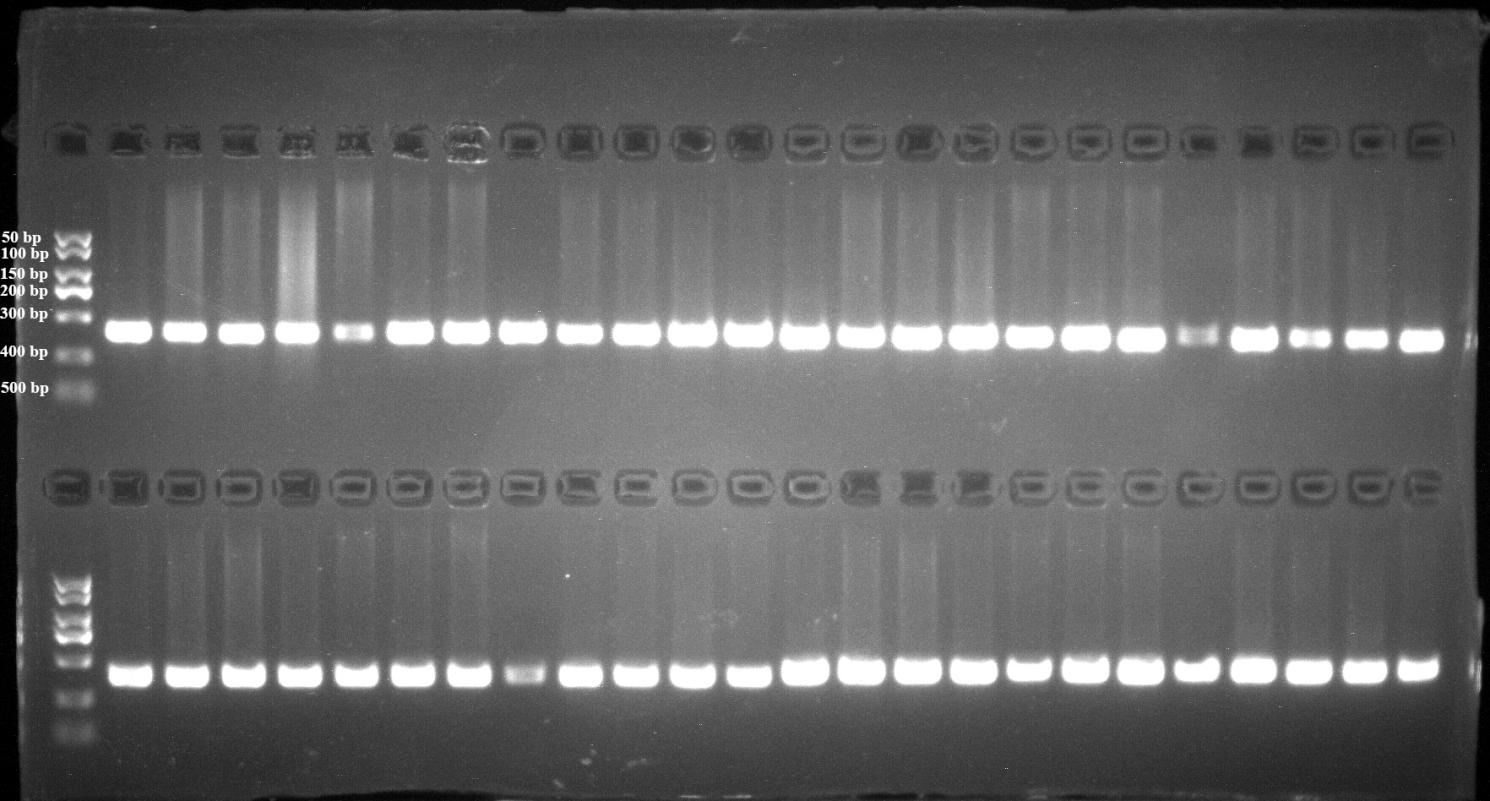


Supplementary Fig. 3. Agarose gel electrophoresis of PCR-amplified *rbcL* region.


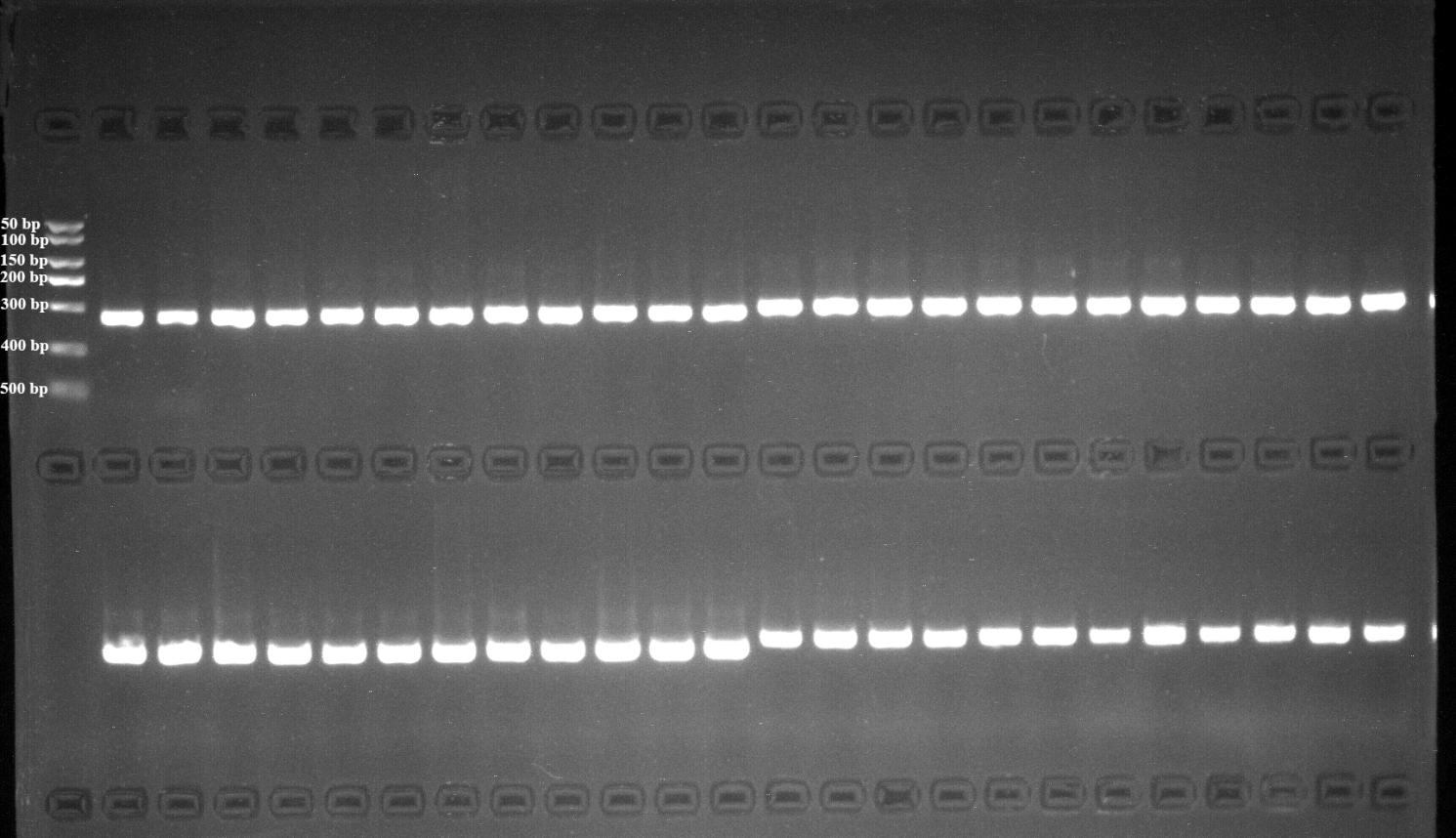


Supplementary Fig. 4. Agarose gel electrophoresis of PCR-amplified *rps16* region.
